# Supplementary figures and images for: Evaluation of polygenic prediction methodology within a reference-standardized framework
Source: PLoS Genet. 2021 May 4;17(5):e1009021. doi: 10.1371/journal.pgen.1009021 (PMC8121285; doi:10.1371/journal.pgen.1009021)

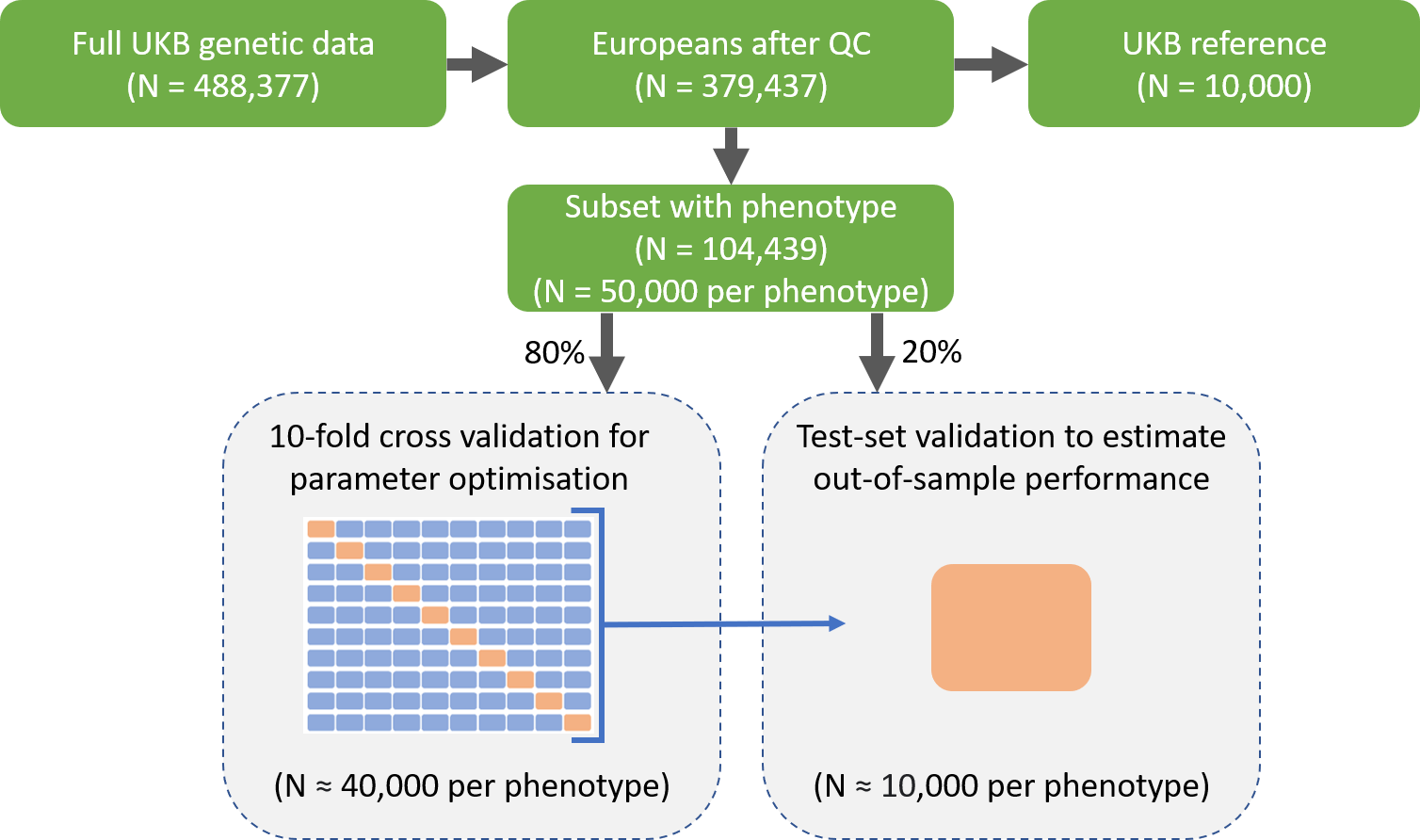

Supplement: S1 Fig — A sample of UKB providing 50,000 observations for each phenotype was identified. The sample was then further split into training (80%) and testing (20%) samples. The training sample used 10-fold cross validation to identify the optimal polygenic scoring parameters and elastic net hyper-parameters. An independent sample of 10,000 European UKB participants was also created to as a reference for polygenic scoring. (PNG) [file pgen.1009021.s001.png]

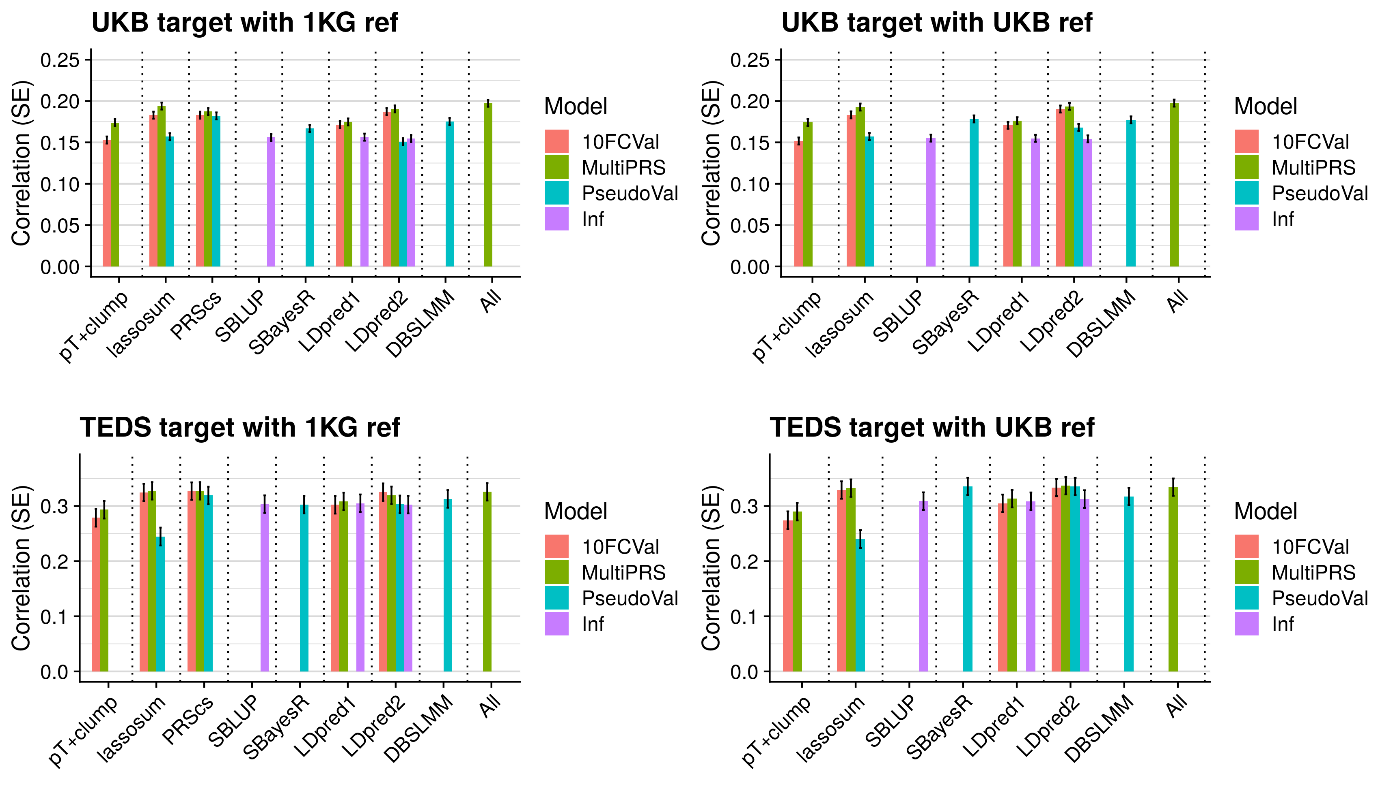

Supplement: S2 Fig — Error bars indicate standard error of correlations for each method. Results are split by the target and reference genotypic data used. Results are 10FCVal bars represent a single polygenic score based on the optimal parameter as identified using 10-fold cross-validation. Multi-PRS bars represent an elastic net model containing polygenic scores based on a range of parameters, with elastic net shrinkage parameters derived using 10-fold cross-validation. PseudoVal bars represent a single polygenic score based on the predicted optimal parameter as identified using pseudovalidation, which requires no tuning sample. Inf represents a single polygenic score based on the infinitesimal model, which requires no tuning sample. (PNG) [file pgen.1009021.s002.png]

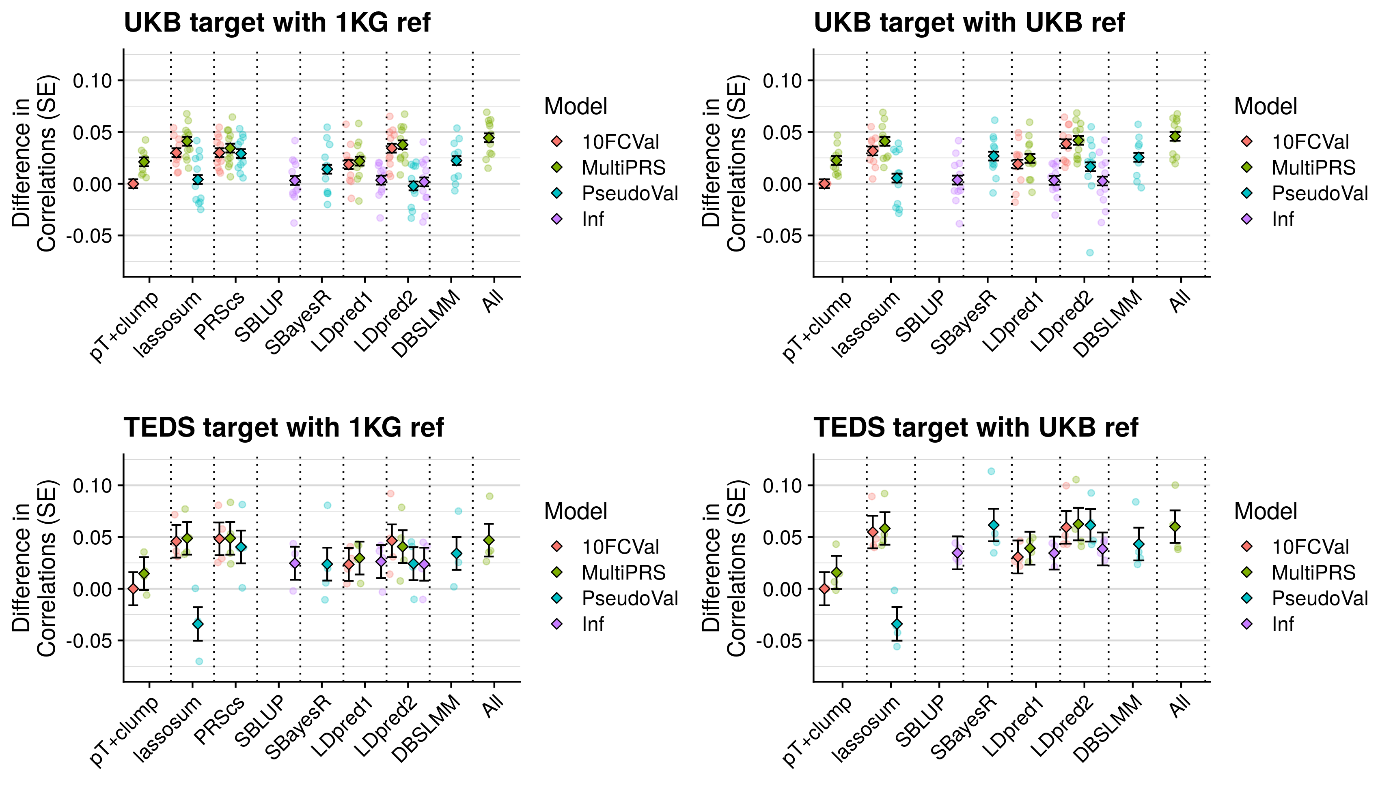

Supplement: S3 Fig — The average difference across phenotypes are shown as diamonds with error bars indicating the standard error, and the difference for each phenotype shown as transparent circles. Results are split by the target and reference genotypic data used. 10FCVal represents a single polygenic score based on the optimal parameter as identified using 10-fold cross-validation. Multi-PRS represents an elastic net model containing polygenic scores based on a range of parameters, with elastic net shrinkage parameters derived using 10-fold cross-validation. PseudoVal represents a single polygenic score based on the predicted optimal parameter as identified using pseudovalidation, which requires no tuning sample. Inf represents a single polygenic score based on the infinitesimal model, which requires no tuning sample. (PNG) [file pgen.1009021.s003.png]

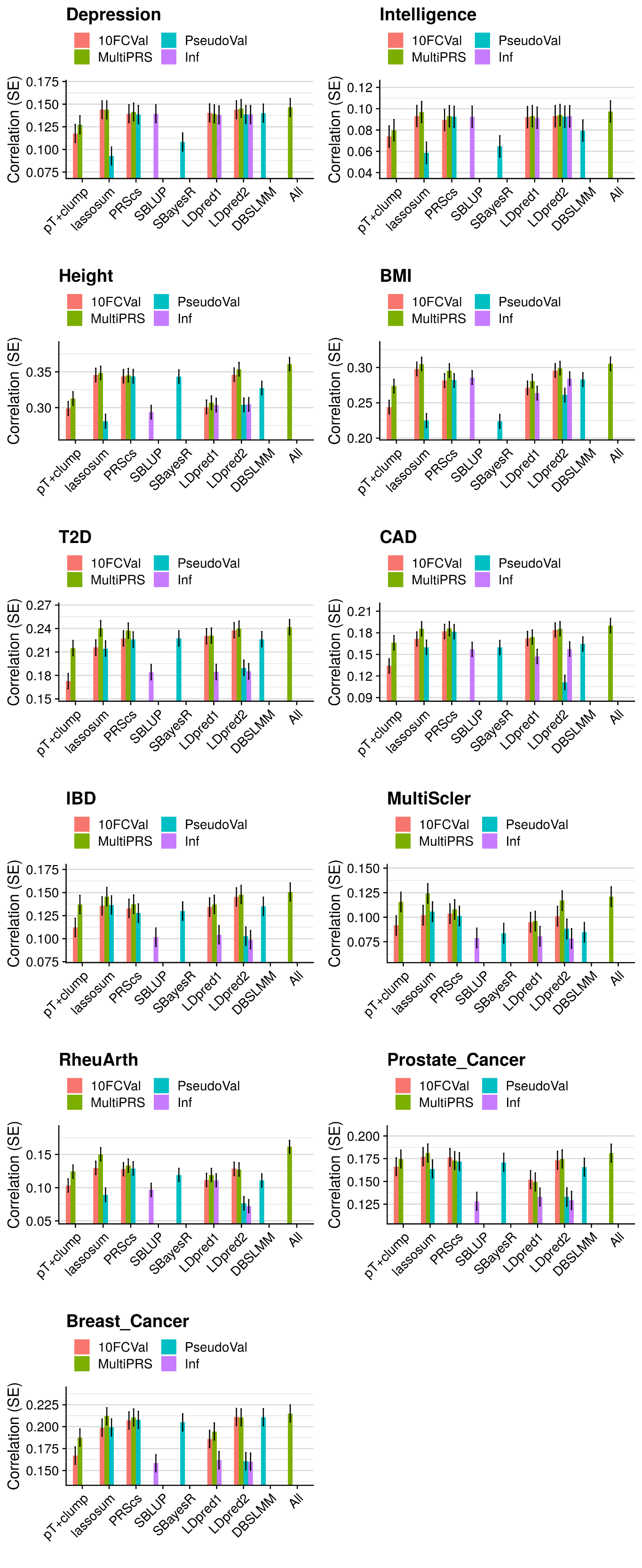

Supplement: S4 Fig — Error bars indicate standard errors. 10FCVal bars represent a single polygenic score based on the optimal parameter as identified using 10-fold cross-validation. Multi-PRS bars represent an elastic net model containing polygenic scores based on a range of parameters, with elastic net shrinkage parameters derived using 10-fold cross-validation. PseudoVal bars represent a single polygenic score based on the predicted optimal parameter as identified using pseudovalidation, which requires no tuning sample. Inf represents a single polygenic score based on the infinitesimal model, which requires no tuning sample. (PNG) [file pgen.1009021.s004.png]

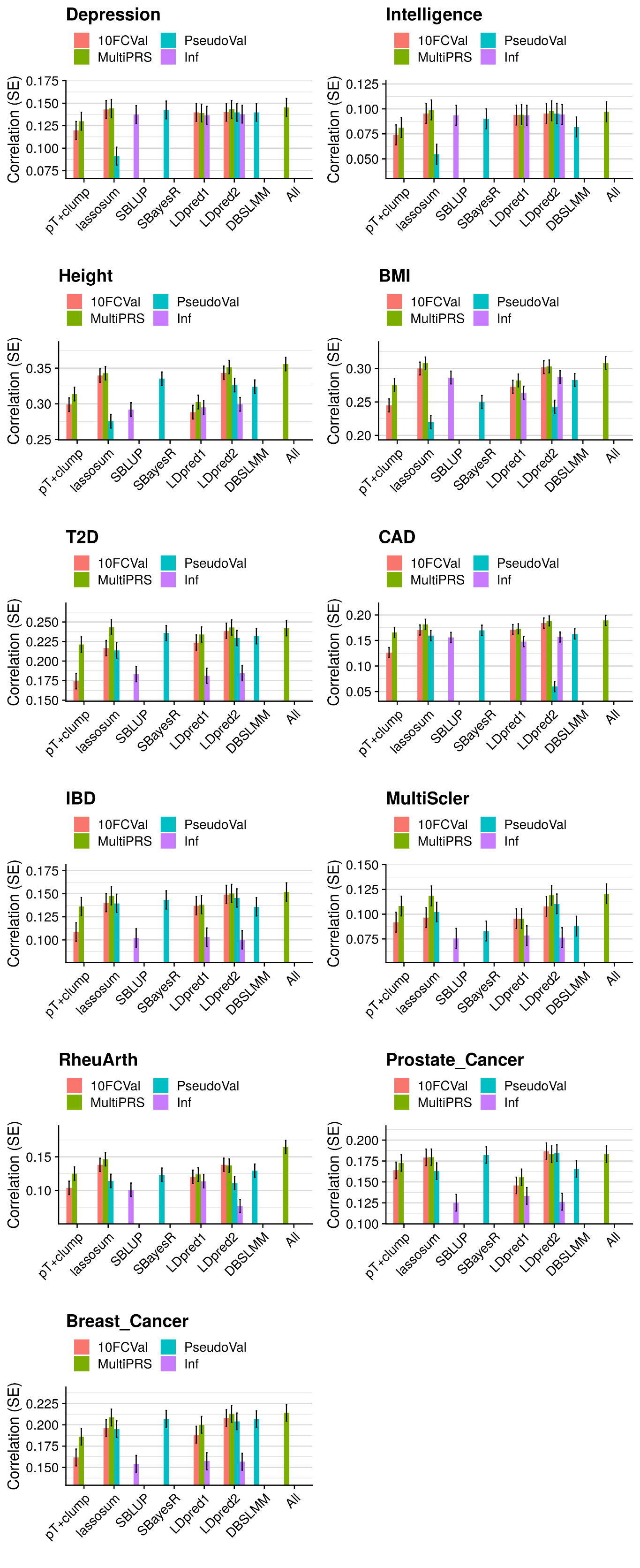

Supplement: S5 Fig — Error bars indicate standard errors. 10FCVal bars represent a single polygenic score based on the optimal parameter as identified using 10-fold cross-validation. Multi-PRS bars represent an elastic net model containing polygenic scores based on a range of parameters, with elastic net shrinkage parameters derived using 10-fold cross-validation. PseudoVal bars represent a single polygenic score based on the predicted optimal parameter as identified using pseudovalidation, which requires no tuning sample. Inf represents a single polygenic score based on the infinitesimal model, which requires no tuning sample. (PNG) [file pgen.1009021.s005.png]

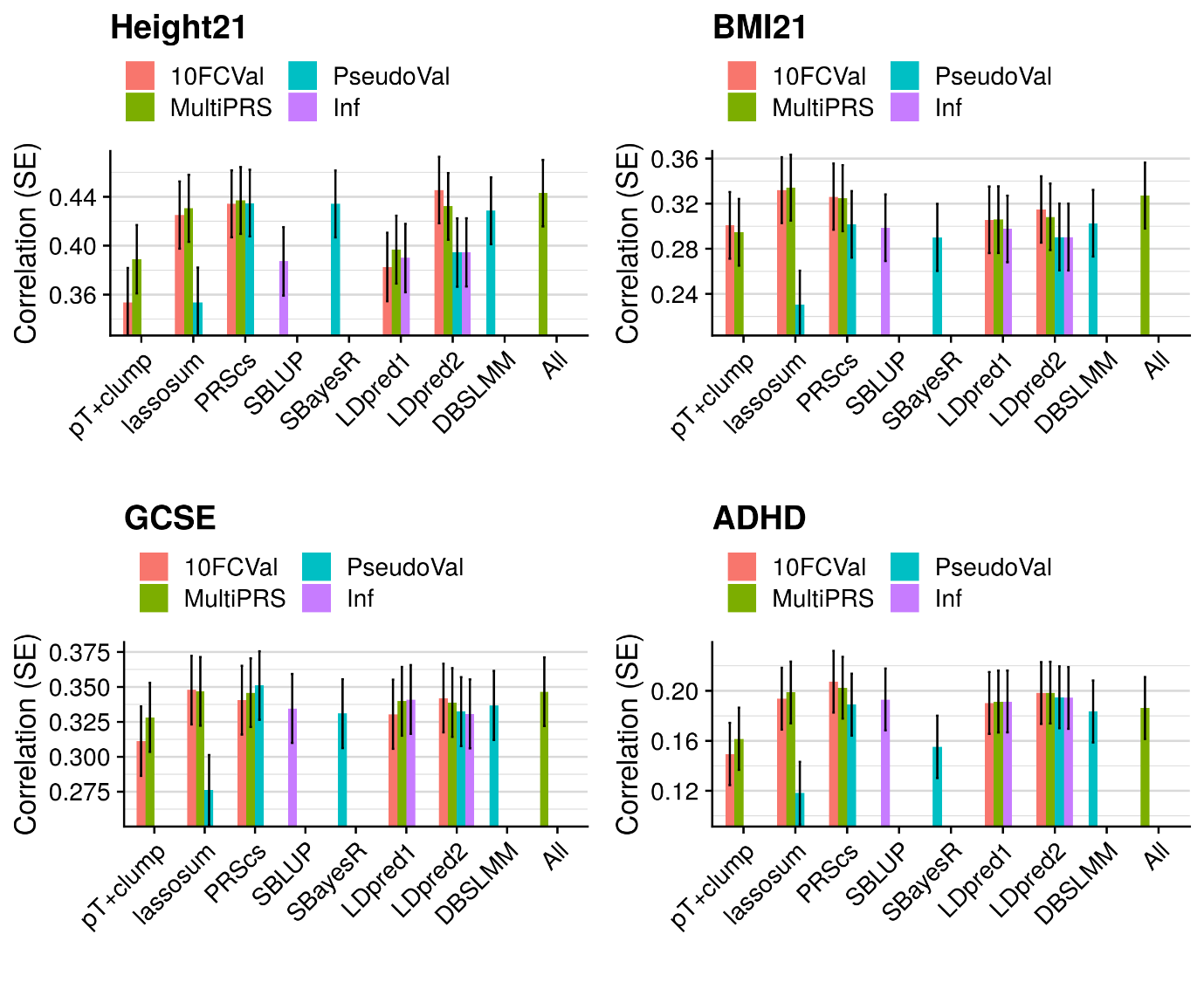

Supplement: S6 Fig — Error bars indicate standard errors. 10FCVal bars represent a single polygenic score based on the optimal parameter as identified using 10-fold cross-validation. Multi-PRS bars represent an elastic net model containing polygenic scores based on a range of parameters, with elastic net shrinkage parameters derived using 10-fold cross-validation. PseudoVal bars represent a single polygenic score based on the predicted optimal parameter as identified using pseudovalidation, which requires no tuning sample.1000G Reference. Inf represents a single polygenic score based on the infinitesimal model, which requires no tuning sample. (PNG) [file pgen.1009021.s006.png]

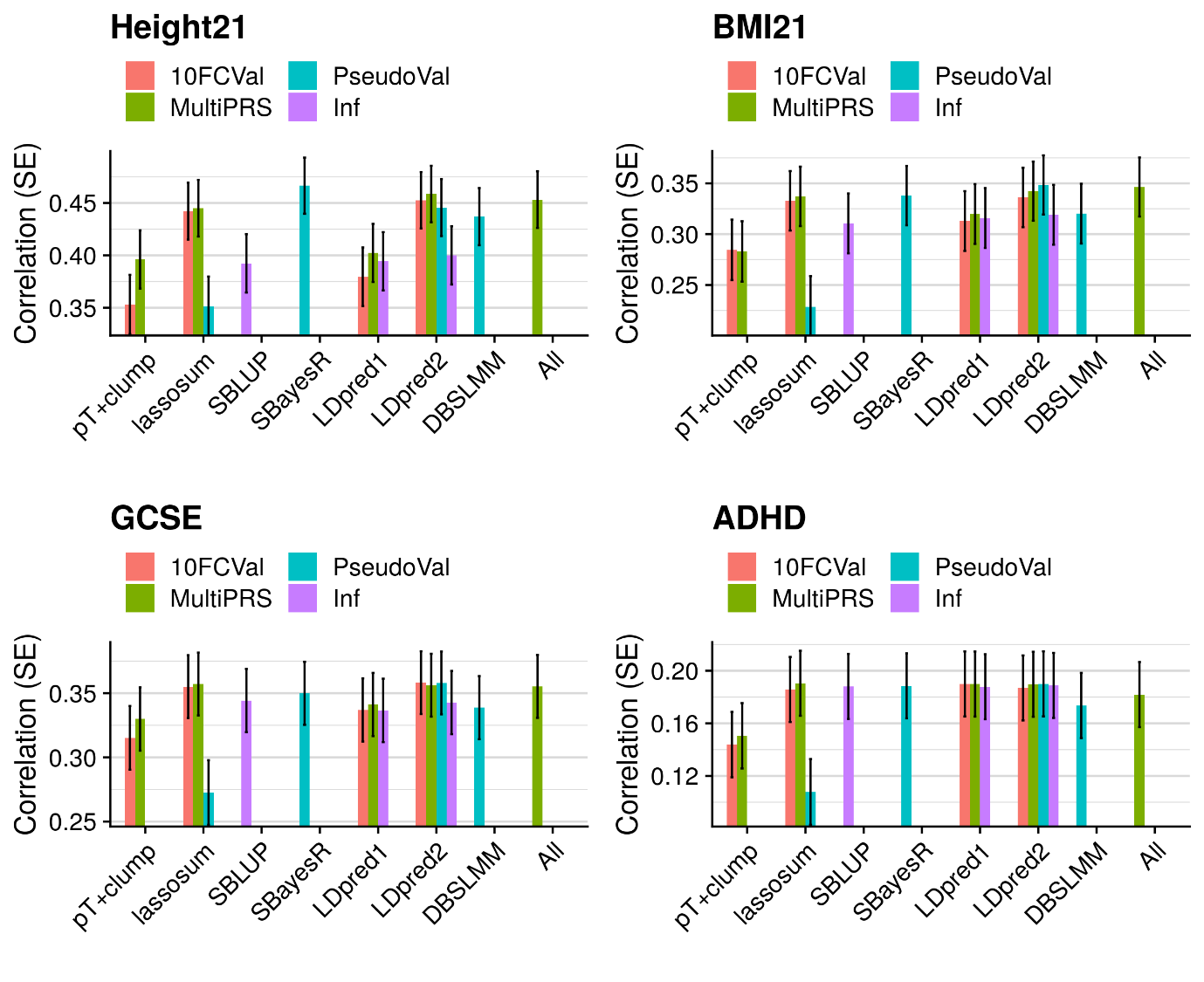

Supplement: S7 Fig — Error bars indicate standard errors. 10FCVal bars represent a single polygenic score based on the optimal parameter as identified using 10-fold cross-validation. Multi-PRS bars represent an elastic net model containing polygenic scores based on a range of parameters, with elastic net shrinkage parameters derived using 10-fold cross-validation. PseudoVal bars represent a single polygenic score based on the predicted optimal parameter as identified using pseudovalidation, which requires no tuning sample. Inf represents a single polygenic score based on the infinitesimal model, which requires no tuning sample. (PNG) [file pgen.1009021.s007.png]

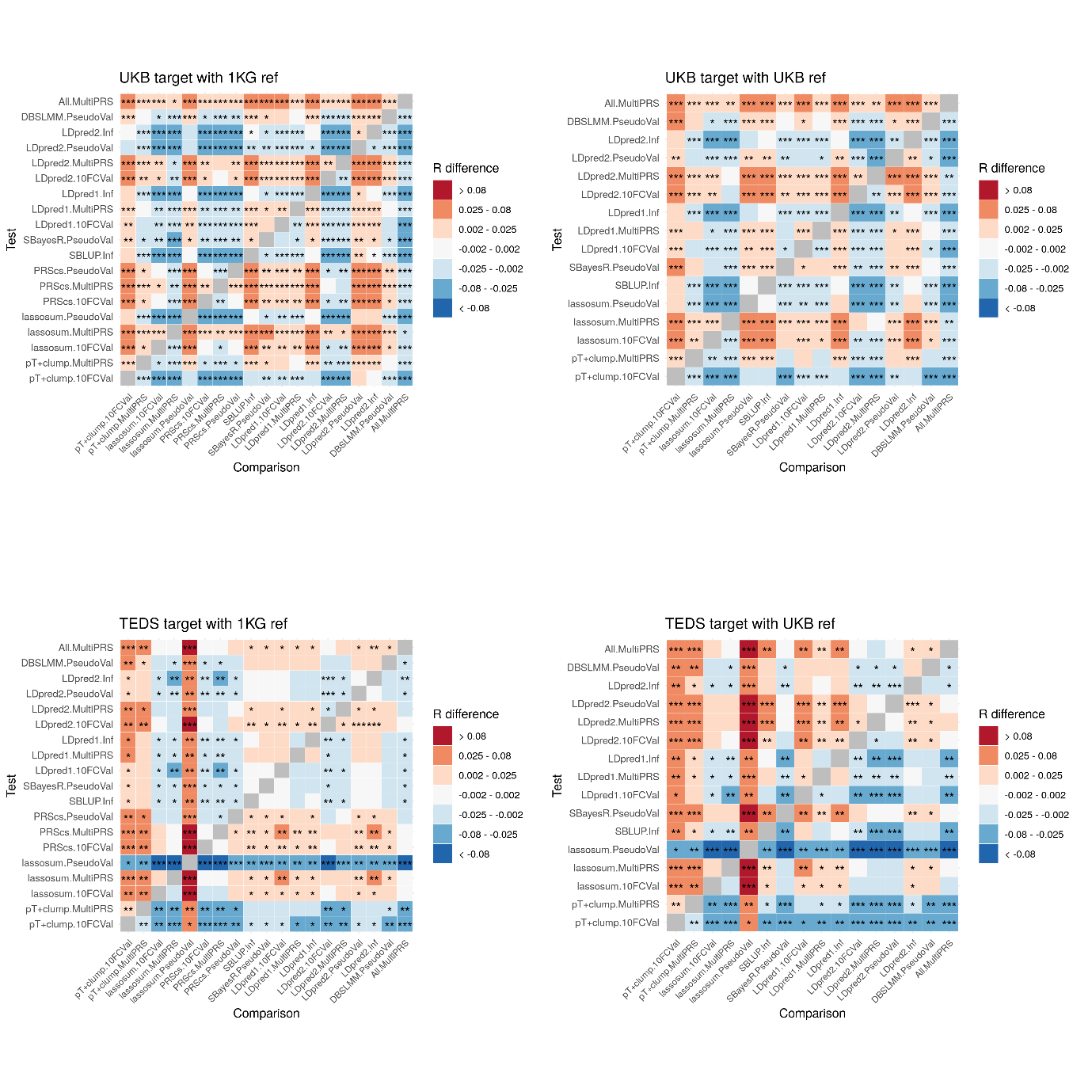

Supplement: S8 Fig — Correlation difference = Test correlation–Reference correlation. Shows only results based on the UKB target sample when using the 1KG reference as other results were highly concordant. * = p<0.05. ** = p<1×10^−3^. *** = p<1×10^−6^. P-values are one-sided. 10FCVal corresponds to a single polygenic score based on the optimal parameter as identified using 10-fold cross-validation. Multi-PRS corresponds to an elastic net model containing polygenic scores based on a range of parameters, with elastic net shrinkage parameters derived using 10-fold cross-validation. PseudoVal corresponds to a single polygenic score based on the predicted optimal parameter as identified using pseudovalidation, which requires no tuning sample. Inf represents a single polygenic score based on the infinitesimal model, which requires no tuning sample. (PNG) [file pgen.1009021.s008.png]

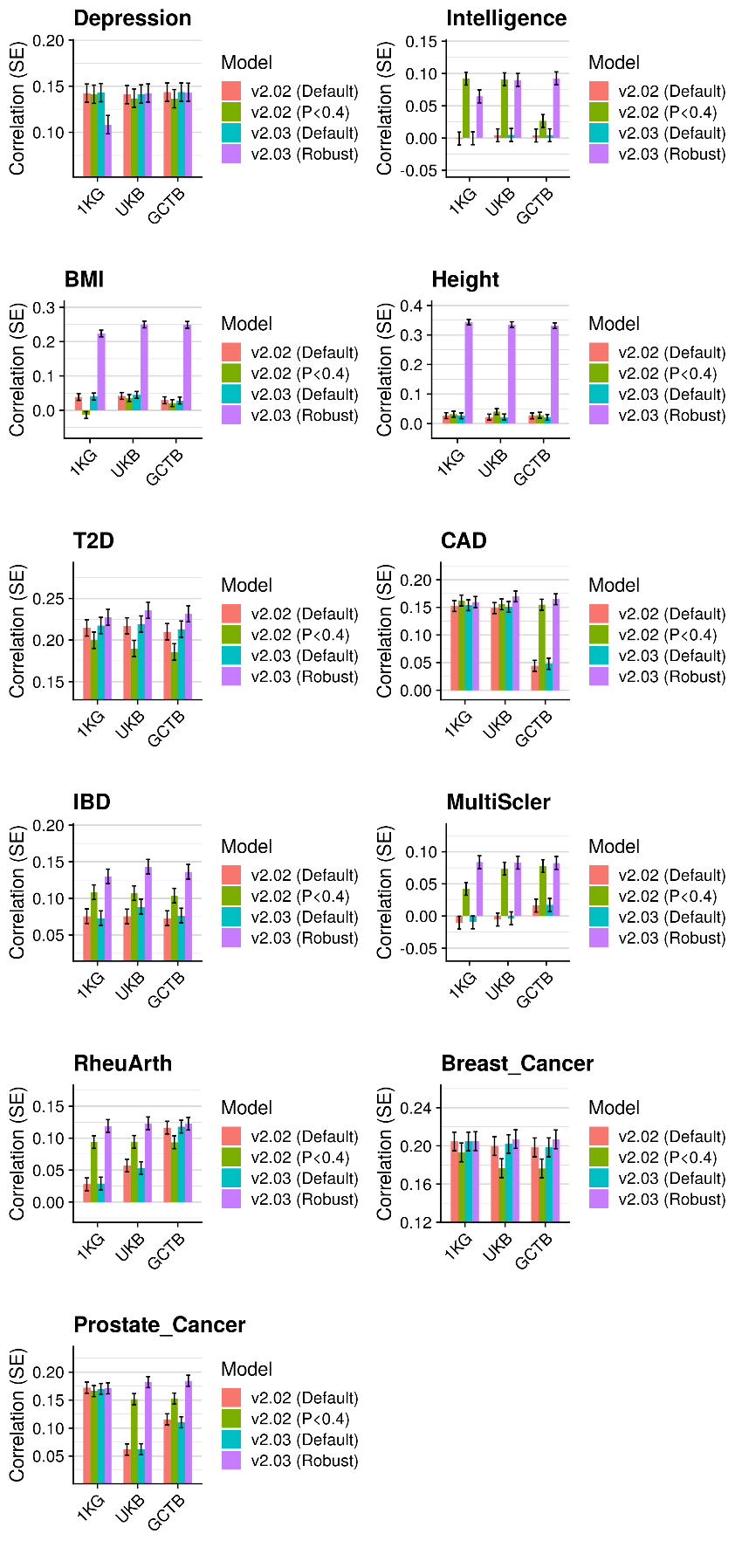

Supplement: S9 Fig — 1KG indicates the reference sample was the European subset of 1000 Genomes. UKB indicates the reference sample was an independent 10K subset of European UKB individuals. GCTB indicates the reference was the GCTB-provided reference data based on a non-independent 50K subset of European UKB individuals. The colour of the bars indicates the version of GCTB used when running SBayesR and which settings were used. Default indicates that default settings were used. P<0.4 indicates only variants with a GWAS p-value <0.4 were retained. Robust indicates that the—robust parameter was specified, forcing robust parameterisation. (PNG) [file pgen.1009021.s009.png]

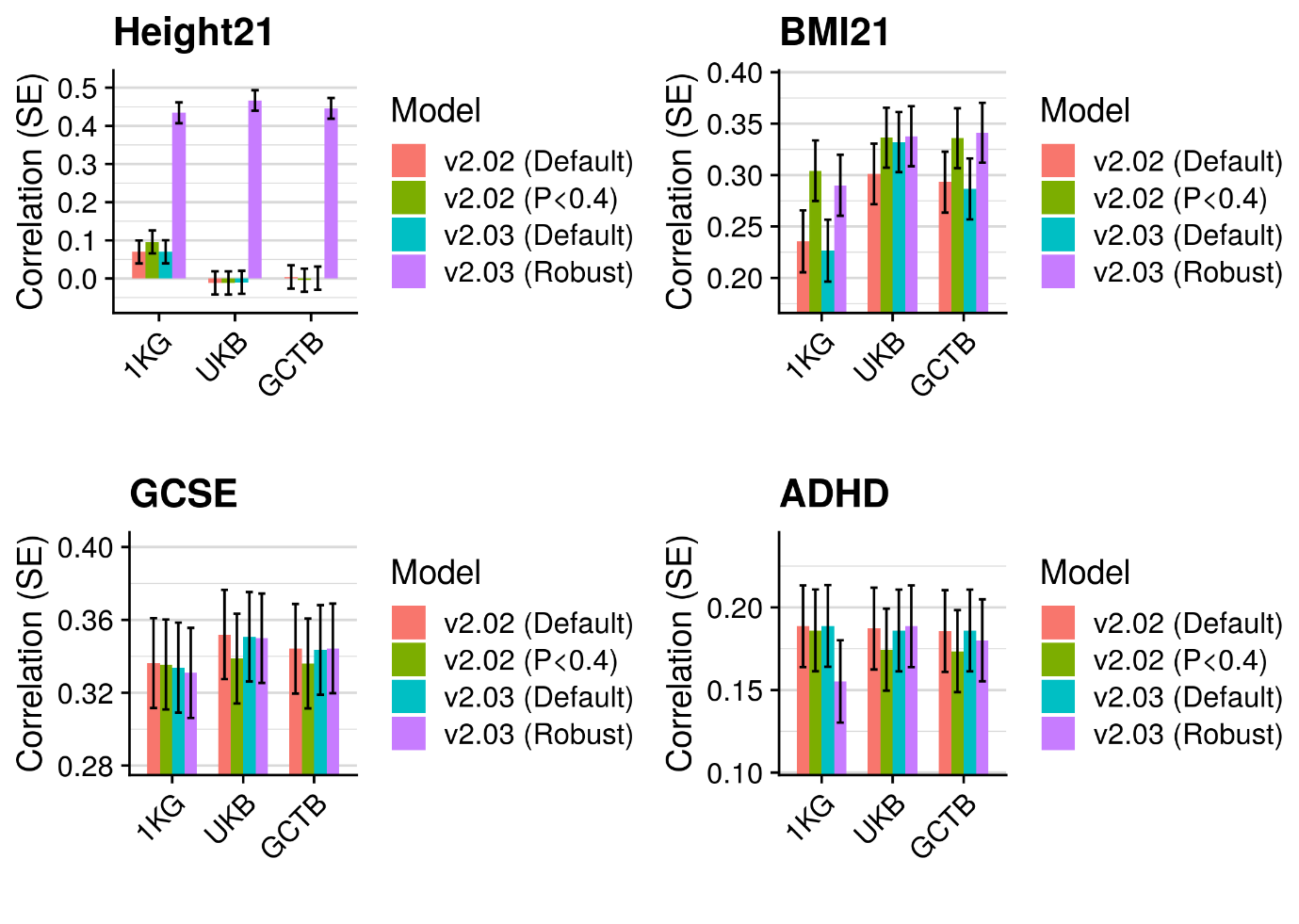

Supplement: S10 Fig — 1KG indicates the reference sample was the European subset of 1000 Genomes. UKB indicates the reference sample was an independent 10K subset of European UKB individuals. GCTB indicates the reference was the GCTB-provided reference data based on a non-independent 50K subset of European UKB individuals. The colour of the bars indicates the version of GCTB used when running SBayesR and which settings were used. Default indicates that default settings were used. P<0.4 indicates only variants with a GWAS p-value <0.4 were retained. Robust indicates that the—robust parameter was specified, forcing robust parameterisation. (PNG) [file pgen.1009021.s010.png]

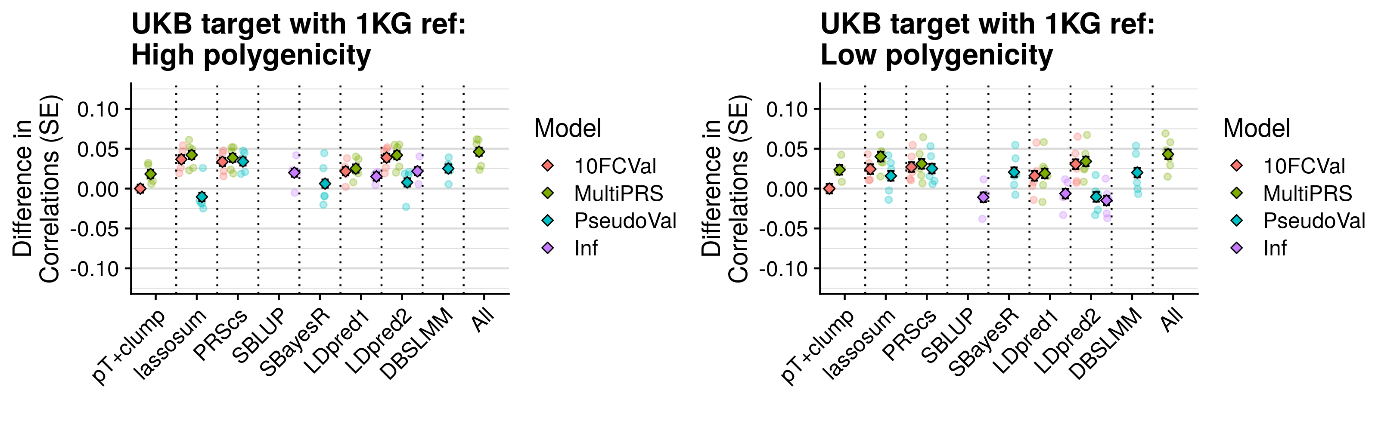

Supplement: S11 Fig — Figure shows average test-set observed-expected correlation difference between the best pT+clump polygenic score and all other methods. The average difference across phenotypes are shown as diamonds with error bars indicating the standard error, and the difference for each phenotype shown as transparent circles. 10FCVal represents a single polygenic score based on the optimal parameter as identified using 10-fold cross-validation. Multi-PRS represents an elastic net model containing polygenic scores based on a range of parameters, with elastic net shrinkage parameters derived using 10-fold cross-validation. PseudoVal represents a single polygenic score based on the predicted optimal parameter as identified using pseudovalidation, which requires no tuning sample. Inf represents a single polygenic score based on the infinitesimal model, which requires no tuning sample. (PNG) [file pgen.1009021.s011.png]

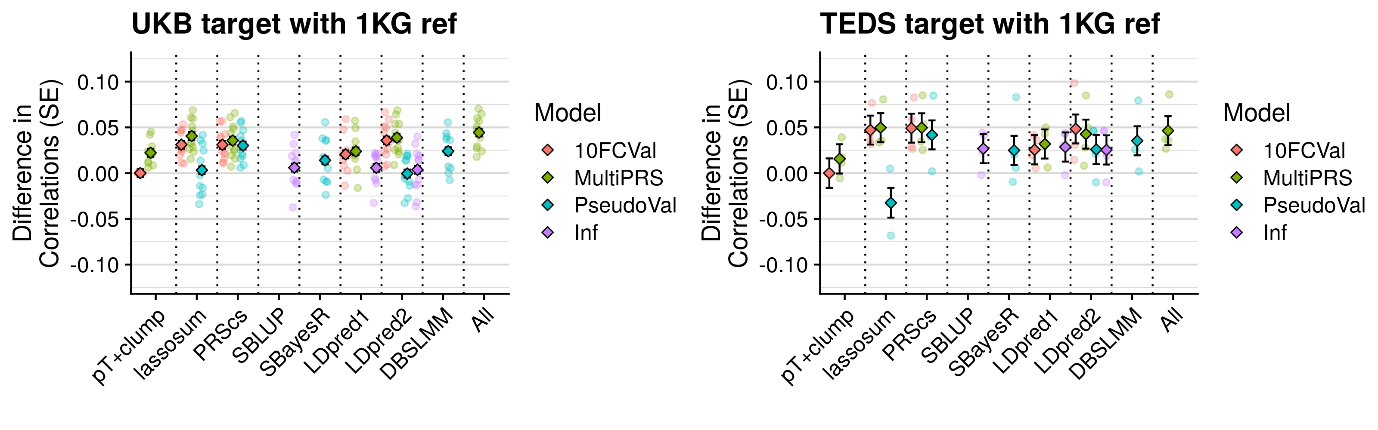

Supplement: S12 Fig — Figure shows average test-set observed-expected correlation difference between the best pT+clump polygenic score and all other methods. The average difference across phenotypes are shown as diamonds with error bars indicating the standard error, and the difference for each phenotype shown as transparent circles. 10FCVal represents a single polygenic score based on the optimal parameter as identified using 10-fold cross-validation. Multi-PRS represents an elastic net model containing polygenic scores based on a range of parameters, with elastic net shrinkage parameters derived using 10-fold cross-validation. PseudoVal represents a single polygenic score based on the predicted optimal parameter as identified using pseudovalidation, which requires no tuning sample. Inf represents a single polygenic score based on the infinitesimal model, which requires no tuning sample. (PNG) [file pgen.1009021.s012.png]

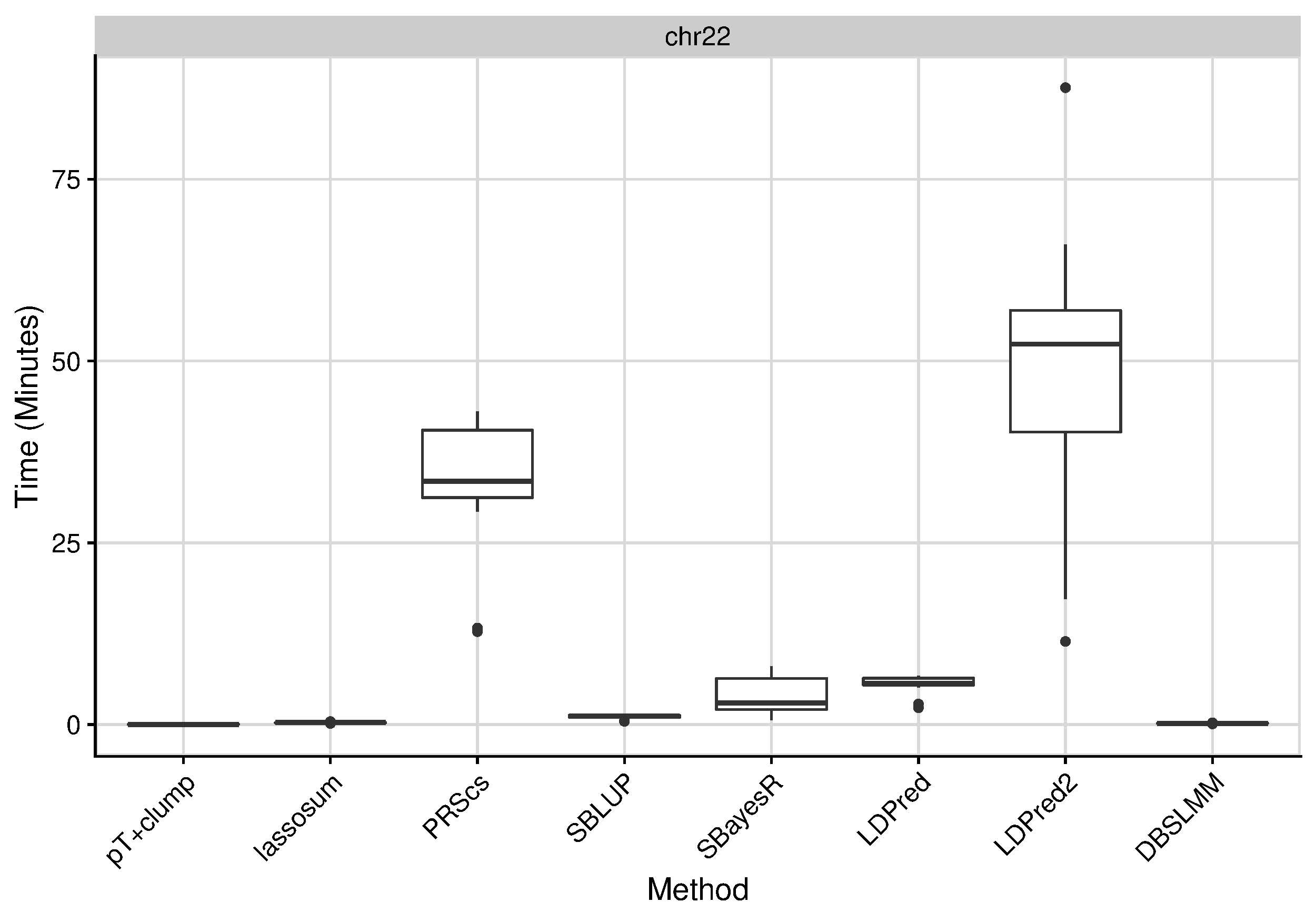

Supplement: S13 Fig — No parallel implementations were used. (PNG) [file pgen.1009021.s013.png]
