# Supplementary material for: Evaluation of polygenic prediction methodology within a reference-standardized framework
Source: PLoS Genet. 2021 May 4;17(5):e1009021. doi: 10.1371/journal.pgen.1009021 (PMC8121285; doi:10.1371/journal.pgen.1009021)
Supplement: S1 Supplementary Text — (DOCX) [file pgen.1009021.s034.docx]

# Supplementary Text

## UKB Outcome definitions

*Depression.* UKB participants were coded as depression cases if they met the Composite International Diagnostic Interview Short Form criteria for lifetime depression which was assessed in the online Mental Health Questionnaire (MHQ) using scoring protocols proposed by Davis et al [1]. Depression cases were screened for indications of schizophrenia or bipolar disorder according to the MHQ. Controls excluded if they show any psychiatric indications according to the MHQ or depression indications according to: ICD-10 diagnoses; endorsement of self-reported depression; endorsement of current antidepressant usage; single or current depression according to the criteria adopted by Smith, et al [2]. Further details of the exclusion criteria have been previously described [3].

*T2D.* Cases were identified based on a combination of hospital episode statistics, using both ICD-9 and ICD-10, the national death register, and self-reported questionnaire data. In order to classify as a case for type 2 diabetes, self-reported type 2 or generic diabetes status was established in the nurse interview and the touchscreen questionnaire. However, participants were only classified as cases when they reported in the questionnaire that they had not been treated with insulin in the first year after diagnosis and had been diagnosed after the age of 35 years. Type 2 diabetes controls did not fulfil these criteria and did not have any other types of diabetes. Further details of the T2D definition have been previously published [4].

*Coronary artery disease (CAD).* Participants who were registered in the hospital in-patient data or the death register to have had ischemic heart diseases, or participants who had coronary revascularization operations were classified as coronary artery disease cases in this study. If participants self-reported those conditions in the nurse interview or the touchscreen questionnaire, they were also considered to have coronary artery disease. Coronary artery disease controls did not fulfil those criteria. Further details of the CAD definition have been previously published [4].

*Autoimmune diseases (IBD, RheuArth, MultiScler):* UKB participants were coded as autoimmune cases if at least two of the following measures were observed: ICD-10 diagnoses from Hospital Episode Statistics; endorsement of self-reported autoimmune diseases; endorsement of prescription medication for the corresponding autoimmune diseases. More than one hospital admission for the respective autoimmune conditions was also sufficient. Controls were excluded if any of the following were observed: Pernicious Anemia, Autoimmune Thyroid Disease, Type 1 diabetes, Multiple Sclerosis, Myasthenia Gravis, Coeliac, Inflammatory Bowel Disease, Hidradenitis Suppurativa, Pemphigoid/Pemphigus, Psoriasis, Ankylosing Spondylitis, Polymyalgia Rheumatica/Giant Cell Arteritis, Psoriatic Arthritis, Rheumatoid Arthritis, Sjögren Syndrome, Systemic Lupus Erythematosus.

*Intelligence* was defined using the Fluid intelligence score variable. Fluid intelligence was assessed using the 13 item UKB Touch-screen Fluid intelligence test [5]. The test measures the capacity to solve problems that require logic and reasoning ability, independent of acquired knowledge. The fluid intelligence variable was derived by UKB as an unweighted sum of the number of correct answers, assigning a score of 0 to unanswered questions.

*Height* was defined using the Standing height variable (Field ID: f.50.0.0).

*BMI* was defined using the Body mass index variable (Field ID: f.21001.0.0).

Breast Cancer and Prostate Cancer were defined using the self-reported illness codes (1044 = prostate cancer, 1002 = breast cancer, Field ID: f.20001).

## TEDS outcome definitions

*Height*: Self-reported height was assessed at age 21.

*BMI*: BMI was calculated as self-reported weight in kilograms divided by height in meters squared (kg/m^2^) at age 21.

*Educational Achievement*: Results for standardized tests taken at the end of compulsory education in the United Kingdom (General Certificate of Secondary Education; GCSE) were obtained for twins at mean age 16.3 years (SD = 0.29) via self-report or parent-report. Grades were coded from 4 (G; the minimum pass grade) to 11 (A*; the highest possible grade), with the U fail grade coded as missing. A composite score was calculated as the arithmetic mean of the compulsory core subjects—Maths, English, and Science. Further information on this definition of Educational Attainment has been previously published [6].

*Attention-Deficit Hyperactivity Disorder (ADHD) Symptoms*: At age 11.5 (SD = 0.69) and 16.3 (SD = 0.69), parents reported on twins’ ADHD symptoms via the Strength and Difficulties Questionnaire [7] hyperactivity subscale (three-point Likert scale) and the Conners’ rating scales (CPRS-R; four-point Likert scale) [8] on hyperactivity and inattention. A composite score was created as the arithmetic mean of the sex and age z-standardized scales. Where ratings were available at one assessment only, this value was used to maximize sample size.

Estimating polygenicity

To investigate whether the polygenicity of the GWAS phenotype affects the relative performance of each polygenic scoring method, we averaged the predictive performance of each method across low polygenicity outcomes and high polygenicity outcomes. Polygenicity was estimated using software called AVENGEME [9], which uses pT+clump polygenic score association results across a range of p-value thresholds to estimate the polygenicity of the GWAS phenotype. We defined a GWAS phenotype as highly polygenic if the estimated proportion of variants with zero effect was <0.96. With this threshold the following outcomes were found to have low polygenicity: T2D, IBD, MultiScler, RheuArth, Prostate Cancer and Breast Cancer.

## SBayesR sensitivity analysis

This study originally used GCTB v2.02 when evaluating SBayesR. We found evidence of poor convergence for several GWAS used in this study, as indicated either by the SBayesR analysis not completing, or by a SNP-based heritability estimate of >1. To avoid convergence issues, we originally restricted the analysis to variants with a per-variant sample size within 3SD of the median sample size. However, for nine of the 12 GWAS, per variant sample size was not reported. As recommended by the SBayesR authors, for GWAS without per-variant sample size the GCTB ‘--impute-n’ option was used (imputes per variant sample size and removes variants with a sample size over 3SD from the median). Furthermore, if there was evidence of poor convergence, the analysis was restricted to variants with a p-value <0.4, as originally recommended by the SBayesR developers.

During our study, a new version of GCTB (v2.03) was released, which included an option to use a more robust parameterisation (--robust) and avoid convergence issues. This robust parameterisation is automatically initiated when the software detects convergence issues. However, when evaluating this new version of GCTB, we found the software did not always detect evidence of convergence issues, and the performance of SBayesR generally improved when the robust parameterisation was forced by setting the --robust option. The performance of SBayesR using GCTB v2.02, with and without restricting to variants with p-value <0.4, and SBayesR using GCTB v2.03, with and without forcing robust parameterisation, are shown in Figs S9-S10).

# References

1. Davis KAS, Coleman JRI, Adams M, Allen N, Breen G, Cullen B, et al. Mental health in UK Biobank–development, implementation and results from an online questionnaire completed by 157 366 participants: a reanalysis. BJPsych open. 2020;6.

2. Smith DJ, Nicholl BI, Breda Cullen DM, Ul-Haq Z, Evans J, Gill JMR, et al. Prevalence and characteristics of probable major depression and bipolar disorder within UK biobank: cross-sectional study of 172,751 participants. PLoS One. 2013;8.

3. Glanville KP, Coleman JRI, Hanscombe KB, Euesden J, Choi SW, Purves KL, et al. Classical human leukocyte antigen alleles and C4 haplotypes are not significantly associated with depression. Biol Psychiatry. 2020;87: 419–430.

4. Fürtjes AE, Coleman JRI, Tyrrell J, Lewis CM, Hagenaars SP. Phenotypic Associations and Shared Genetic Etiology between Bipolar Disorder and Cardiometabolic Traits. medRxiv. 2020.

5. Sudlow C, Gallacher J, Allen N, Beral V, Burton P, Danesh J, et al. UK biobank: an open access resource for identifying the causes of a wide range of complex diseases of middle and old age. PLoS Med. 2015;12.

6. Krapohl E, Rimfeld K, Shakeshaft NG, Trzaskowski M, McMillan A, Pingault J-B, et al. The high heritability of educational achievement reflects many genetically influenced traits, not just intelligence. Proc Natl Acad Sci. 2014;111: 15273–15278.

7. Goodman R. The Strengths and Difficulties Questionnaire: a research note. J child Psychol psychiatry. 1997;38: 581–586.

8. Conners CK. Conners’ Rating Scales-revised: Technical Manual:[instruments for Use with Children and Adolescents]. MHS; 1998.

9. Palla L, Dudbridge F. A fast method that uses polygenic scores to estimate the variance explained by genome-wide marker panels and the proportion of variants affecting a trait. Am J Hum Genet. 2015;97: 250–259.

10. Lloyd-Jones LR, Zeng J, Sidorenko J, Yengo L, Moser G, Kemper KE, et al. Improved polygenic prediction by Bayesian multiple regression on summary statistics. Nat Commun. 2019;10: 1–11.
