# Supplementary material for: Evaluation of polygenic prediction methodology within a reference-standardized framework
Source: PLoS Genet. 2021 May 4;17(5):e1009021. doi: 10.1371/journal.pgen.1009021 (PMC8121285; doi:10.1371/journal.pgen.1009021)
Supplement: Attachment — Submitted filename: Response letter V3.docx [file pgen.1009021.s035.docx]

Dear PLOS Genetics editors,

We thank you for sending our manuscript for review and providing clear guidance. We also thank the reviewers for their helpful comments.

We have responded to each of the Editorial and Reviewer comments below (highlighted in blue)

**Editorial comments:**

Reading through the reviews, a specific comment that occurred to me before I sent this paper out for review and that was confirmed by reviewers is the need to report more standard metrics for binary outcomes (AUC/OR per SD). I mention this because one of my first reaction was to line up these results with my personal intuition, and while I appreciate your argument that a corresponding value can be estimated in theory, it remains a hurdle. So that would be a meaningful improvement. A reviewer noted that not all existing methods are presented but that is understandable of course- there are simply too many. As an editor, I will not require that you extend a lot the set of methods included but please try to consider that comment if you can. On the other hand, a small number of additional disease traits (breast cancer for example?) would be helpful. The cancer space is of particular interest and should be highlighted.

More generally, the reviewers have made many suggestions for improvements and additional comparisons.  We won't expect you to fully meet every such request but we would like to see some substantial improvements in a revised version - we leave to you to choose the most important additions that can be made to improve the manuscript within a reasonable time frame.

*Response:*

Thank you for this clear guidance.

To enable readers to interpret the results more easily, we have added the following columns to Tables S6-S7: Odds ratio per SD, Upper and lower 95% confidence intervals of the OR, area under the ROC curve (AUC), and the R2 on the liability scale.

We agree it is important to compare a wider range of methods. As a result, we have provided a richer comparison by including comparison with two additional methods, DBSLMM and LDPred2. We have included discussion of the other methods suggested by the reviewer. We have not included MegaPRS as the associated study is not yet published. We have not included AnnoPred, or ldpred-funct, as these methods include functional annotations when deriving the polygenic score and are considered somewhat distinct from other methods. We have not included NPS or SCT as these methods are again somewhat distinct as they are unable to generate a polygenic score without a training sample with the desired phenotype. Furthermore, previous comparison of NPS and SCT indicates these methods are unlikely to improve prediction over the other methods included ([https://academic.oup.com/bioinformatics/advance-article/doi/10.1093/bioinformatics/btaa1029/6039173#221959715](about:blank#221959715), [https://www.sciencedirect.com/science/article/pii/S0002929720301518](about:blank)) .

To increase the number of outcomes of low polygenicity in the study, we have now included two of the suggested outcomes, prostate cancer and breast cancer. AVENGEME estimates (Table S21) confirm that we have a good balance of high and low polygenicity outcomes in our study.

Reviewer #1:

In this report, Pain et al. set out to benchmark various polygenic score algorithms as well as two reference panels across a range of 13 traits. They determine that on average: (i) all scores outperformed pruning and thresholding based approaches; (ii) PRScs delivered performance comparable to other approaches but dit not require a tuning set; (iii) use of a polygenic score based on an elastic net tended to outperform other strategies.

I do think that this approach will be of interest to the general genetics community.

* Please clarify - multiPRS for each algorithms are based on combining multiple scores derived from a single algorithm?

*Response:*

Yes, the term multiPRS refers to a model containing multiple polygenic scores derived using a single method. This has been clarified in text as follows:

“In addition to selecting the single ‘best’ parameter for polygenic scoring, elastic net models were derived containing polygenic scores based on a range of parameters for each method, with elastic net shrinkage parameters derived using 10-fold cross-validation (Multi-PRS). The number of scores generated by each method, which were included in the multi-PRS model, are shown in Table 2.”

* Can score performance be improved using an elastic net of scores using multiple different algorithms do even better?

*Response:*

Good question. We have now included comparison to a model containing all polygenic scores from all methods. On average, combining polygenic scores from a range of methods does not significantly improve prediction over the MultiPRS lassosum method. Given the considerable computation time and analyst time required to run all methods, we do not advise this combined method. Figures including the ‘All’ method have been updated throughout. We describe this addition in the methods, results and discussion as follows:

Methods: “In addition, we tested whether combining polygenic scores from all methods in an elastic net model improved prediction. This combined model is referred to the ‘All’ model.”

Results: “On average, the ‘All’ method, combining polygenic scores across polygenic scoring methods did not provide a statistically significant improvement over the single best method (multi-PRS lassosum).”

Discussion: “Modelling polygenic scores from multiple methods did not significantly improve prediction over the single best method.”

* I think correlation is used as a surrogate for accuracy, but I wonder if additional metrics should also be reported (e.g. a measure of discrimination for binary phenotypes or OR/SD commonly used), can get ideas from the Wand et al. preprint one polygenic score reporting standards from ClinGen

*Response:*

To enable readers to interpret the results more easily, we have added the following columns to Tables S6-S7: Odds ratio per SD, Upper and lower 95% confidence intervals of the OR, area under the ROC curve (AUC), and the R2 on the liability scale.

* For P&T, what r2 value(s) were used?

*Response:*

LD-based clumping was performed using an R2 threshold of 0.1, and window of 250kb. This information has now been added to Table 2.

* Can authors comment on general principle of being able to predict which approach might work the best based on heritability, polygenicity, etc.? e.g. autoimmune diseases have a very different architecture so could be optimized using a different approach.

*Response:*

Good question. We have estimated polygenicity for each phenotype using the AVENGEME method, split the phenotypes into high and low polygenicity groups, and then estimated the average performance of methods within these two groups. All TEDS phenotypes were highly polygenic, so the comparison across high and low polygenicity is based on UK Biobank phenotypes alone. A table listing the polygenicity estimate of each phenotype is shown in Table S21. Figure S11 shows the average performance of each method split by polygenicity. In summary, the relative performance of each method was similar across high and low polygenicity outcomes, but infinitesimal models performed better for high polygenicity phenotypes.

This addition is described as follows:

Methods: “In addition to averaging results across all phenotypes, we estimate the average performance of methods within high and low polygenicity phenotypes. The polygenicity of phenotypes was estimated using AVENGEME [36] (more information in Supplementary Material).”

Results: “The relative performance of all methods and modelling approaches was similar across low and high polygenicity phenotypes (Figure S11). Infinitesimal model-based polygenic scores performed better for high polygenicity phenotypes. Estimates of polygenicity for each phenotype are shown in Table S21.”

Supplementary material – Estimating polygenicity: “To investigate whether the polygenicity of the GWAS phenotype affects the relative performance of each polygenic scoring method, we averaged the predictive performance of each method across low polygenicity outcomes and high polygenicity outcomes. Polygenicity was estimated using software called AVENGEME [9], which uses pT+clump polygenic score association results across a range of p-value thresholds to estimate the polygenicity of the GWAS phenotype. We defined a GWAS phenotype as highly polygenic if the estimated proportion of variants with zero effect was <0.98. With this threshold the following outcomes were found to have low polygenicity: IBD, MultiScler, RheuArth, Prostate Cancer and Breast Cancer.”

Table S21: AVENGEME Estimates of Polygenicity

Figure showing PRS method performance split by polygenicity

* For lassosum, is it appropriate to have a set lambda (as opposed to letting the software choose one as done in glimnet R package)?

*Response:*

We used the default lambda values specified by the developers of lassosum. It is possible the lassosum hyperparameter grid search is suboptimal, though increasing the grid search will increase computation time of the method. A larger parameter grid search could improve the performance of all methods, at the cost of additional computation resources, so our study relies on the default and recommended parameters for each method.

* I found Figure 3 quite confusing and nonintuitive, would encourage an alternate presentation style.

*Response:*

Thank you for highlighting this point. We have modified the figure and the figure legend to improve the interpretability.

Figure 3 legend: “Pairwise comparison between all methods, showing average test-set observed-expected correlation difference between all methods with significance value. Correlation difference = Test correlation – Comparison correlation. Red/orange coloring indicates the Test method (shown on Y axis) performed better than the Comparison method (shown on X axis). Shows only results based on the UKB target sample when using the 1KG reference as other results were highly concordant. *=p<0.05. **=p<1×10-3. ***= p<1×10-6. P-values are two-sided. 10FCVal represents a single polygenic score based on the optimal parameter as identified using 10-fold cross-validation. Multi-PRS represents an elastic net model containing polygenic scores based on a range of parameters, with elastic net shrinkage parameters derived using 10-fold cross-validation. PseudoVal represents a single polygenic score based on the predicted optimal parameter as identified using pseudovalidation, which requires no tuning sample. Inf represents a single polygenic score based on the infinitesimal model, which requires no tuning sample.”

* Please clarify how scores will be shared with research community.

*Response:*

We will return the individual-level polygenic scores generated in this study to UKB and TEDS for other researchers to use. As stated in the manuscript, the GWAS summary statistics are all publicly available and analysis code is available through github [https://github.com/opain/GenoPred]

Reviewer #2:

This manuscript sets out to compare several methods for deriving polygenic
scores, across 13 phenotypes, in the UK Biobank and TEDS study. Several tuning
strategies are compared (10-fold cross-validation, pseudovalidation). With the
large number of polygenic score methods today, it is useful for the human
genetics community to benchmark which approaches are more promising.

Overall, this manuscript is a timely and useful addition to the literature. At
the same time, there are many methods being compared across different phenotypes
and often I found it difficult to follow the logic of the analysis and what
message each key result was trying to convey. In addition, some of the key
concepts (e.g., what pseudovalidation means for each method) aren't explained,
making the results hard to understand, especially for readers who aren't
experts in PRS.

Major comments:
===============

* It's not clear whether the terminology of 'reference standardized approach'
narrowly refers to the use of the same LD reference panels and HapMap3 SNPs to
enable consistent evaluation of methods, or to a wider conceptual framework for
precision medicine; this really needs clarification. If it's only the former,
then it's simply good scientific practice to benchmark tools consistently and is
fine as is (with clarification). But if it's the latter (e.g., as hinted in
Discussion pg 22, line 453), then it should be acknowledged that this is only
the first step in such an endeavour and more complex issues will arise, e.g.,
matching the target sample to the best reference sample, admixture, absolute
risk and calibration, etc.

*Response:*

We propose the reference standardised approach to be advantageous both for research and precision medicine. You raise several other important issues that arise when using polygenic scores for personalised medicine, several of which we are investigating currently. We believe the reference standardised approach, can be extended to accommodate these additional considerations and we have added discussion to this point as follows:

“Fifth, we present the reference standardized approach as a conceptual framework for implementing polygenic scores in a clinical setting. However, several additional issues will need to be addressed before they can be used in a clinical setting, such as assigning individuals to the optimal reference population, the presence of admixture, and translating relative polygenic scores into absolute terms.”

* What's the reasoning behind limiting the sample size to n=50,000 for all
phenotypes?

*Response:*

The reasoning was that a sample size of 50,000 individuals provided sufficient statistical power to detect meaningful differences in polygenic score performance and substantially reduced the computation burden of these analyses.

* Figure 1: What is the difference between the boxes 'Pre-imputed genotype data'
and 'Observed genome-wide genotype data'?

*Response:*

These labels indicate that some genome-wide genetic datasets only include observed/genotyped variants and imputation is therefore required, whereas other datasets have already been imputed and so imputation is not required. In our study we are using research datasets which have been imputed in advance. In contrast, if using data from a clinical setting or direct-to-consumer testing, the data may not have already been imputed. I have clarified this point in the figure legend as follows:

“Pre-imputed genotype data = Indicates the observed genotype data has already been imputed; Observed genome-wide genotype data = Indicate the observed genotype data has not been imputed, and therefore requires imputation.”

* An illustration of the actual study design (e.g., the split of UKB into
various reference/tuning/testing subsets etc, and sample sizes in each) would be
very useful as a supplementary figure, I found it difficult to follow which
dataset went into which analysis.

*Response:*

OK. I have created added a Figure S1, which is a schematic representation of the study design.

Supplementary Figure 1 legend: “Schematic diagram showing UKB was split into reference, training and testing samples. A sample of UKB providing 50,000 observations for each phenotype was identified. The sample was then further split into training (80%) and testing (20%) samples. The training sample used 10-fold cross validation to identify the optimal polygenic scoring parameters and elastic net hyper-parameters. An independent sample of 10,000 European UKB participants was also created to as a reference for polygenic scoring.”

* Can you briefly explain pseudovalidation, and why you consider SBLUP/SBayesR
as methods that don't require cross-validation? LDpred has a single parameter
too, yet it was used in cross-validation.

*Response:*

Pseudovalidation is the term used by the lassosum authors to describe the process of predicting the optimal shrinkage parameter without testing a range of parameters in an independent tuning sample. This is analogous to the fully Baysian mode of PRScs, and the ‘auto’ mode of LDPred2. There is also no requirement for a tuning sample when using SBLUP, SBayesR, DBSLMM, or LDPred1/2 infinitesimal model, so I thought it would be helpful to describe these methods using the same term. However, with the addition of LDPred2 which has both an infinitesimal mode and pseudovalidation (‘auto’) mode, we have decided to clearly distinguish methods using an infinitesimal model (SBLUP, LDPred1/2 infintesimal), and those that have a formal pseudovalidation procedure (PRScs, lassosum, SBayesR, LDPred2-auto, DBSLMM). This distinction is made throughout the manuscript, though given both pseudovalidation and infinitesimal approaches do not require a tuning sample, we describe these methods in parallel in the results and discussion. We provide an explanation of this decision in the manuscript as follows:

Introduction: “In addition, lassosum, PRScs and LDPred2 provide a pseudovalidation approach, whereby a single optimal shrinkage parameter is estimated based on the GWAS summary statistics alone, and therefore do not require a tuning sample. SBayesR and DBSLMM can be considered pseudovalidation approaches as they also do not require a tuning sample to identify optimal parameters. Another approach to derive polygenic scores is to assume an infinitesimal model, as is done by SBLUP and the infinitesimal models of LDPred1 and 2 [16]. Similar to pseudovalidation approaches, no tuning sample is required when assuming an infinitesimal model.”

Methods: “Pseudovalidation was performed using the pseudovalidate function in lassosum, the fully-Bayesian approach in PRScs, the auto model in LDPred2. SBayesR and DBSLMM by default estimate the optimal parameters and are therefore considered pseudovalidation methods. Methods assuming an infinitesimal model were SBLUP and the infinitesimal models of LDPred1 and 2.”

Results: “Pseudovalidation and infinitesimal models do not require a tuning sample and their results are therefore described in parallel. Of the methods providing a pseudovalidation and/or infinitesimal approach…”

* For the summary stats, is any MAF cutoff used, and are the same exact SNPs
used by all methods across all summary stats? e.g., LDpred v1 by default filters
out SNPs with MAF<1% and/or palindromic SNPs, but other methods may not.

*Response:*

Thank you for raising this point. Previously GWAS summary statistics were only filtered using the LDSC munge_sumstat.py script. However, this led to a loss of information, such as SE, and we only filtered SNPs by MAF if MAF information was present. As the reviewer says, some methods also filter by reference allele frequency estimates. To avoid this discrepancy across methods we reanalysed, using a more complete GWAS quality control, described as follows in the Methods section:

“GWAS summary statistics underwent quality control to extract HapMap3 variants, remove ambiguous variants, remove variants with missing data, flip variants to match the reference retain variants with a minor allele frequency (MAF) > 0.01 in the European subset of 1KG Phase 3, retain variants with a MAF > 0.01 in the GWAS sample (if available), retain variants with a INFO > 0.6 (if available), remove variants with a discordant MAF (>0.2) between the reference and GWAS sample (if available), remove variants with p-values >1 or </=0, remove duplicate variants, remove variants with sample size >3SD from the median sample size (if per variant sample size is available).”

Results throughout have been updated. There are no changes to the results.

* For the MultiPRS, it's not clear what kind of PRS went into it. Was it only
P+T based PRS or others as well? How many scores?

*Response:*

The term multiPRS refers to a model containing multiple polygenic scores derived using a single method. The number of scores included for each method is indicated within Table 2. This has been clarified in text as follows:

“The number of scores generated by each method, which were included in the multi-PRS model, are shown in Table 2.”


* Fig 2B, the caption and legend mention diamonds, but the actual figure shows
little stars, are they referring to the same thing?

*Response:*

Figure 2B does use diamonds to indicate the point estimate, however the standard errors are so small that the symbol appears to be a star. I have modified the figure to make this clearer.

* More of a comment / Discussion item, the cross-validation (including the
MultiPRS) will be affected by the number of cases in the training set, which is
quite small for some phenotypes in the UKB (e.g., multiple sclerosis), but isn't
really an inherent 'biological' feature of that disease/phenotype but more of a
UKB limitation.

*Response:*

We agree that as the training sample size decreases, the validation procedures ability to identify the optimal parameter will be reduced. This does not appear to be the case for UKB and TEDS phenotypes used here, so we have decided not to include this point in the discussion.

* Figure 3: It's not clear what this figure is trying to convey. Does Reference
mean the n=10,000 UKB dataset? This should be explained in the figure or the
caption. Also the caption says 'For columns, red/orange coloring indicates the
Test method performed better than the Test method (horizontal)', do you mean
'performed better than the Reference method'?

*Response:* Please see response to Reviewer 1, page 5

Minor comments:
===============

* For the UK Biobank, does 'European ancestry' here mean the British White
subset or is it a wider definition?

*Response:*

We describe how Europeans were identified in UKB as follows:

“This study retained individuals that were of European ancestry based on 4-means clustering on the first 2 principal components provided by the UKB (self-reported ancestry was not used),…”

As a part of the reference standardised pipeline, further participants are excluded if they were more than 3SD from the European mean of the first 100 reference projected PCs, as described in the methods:

“Only individuals of European ancestry were retained for polygenic score analysis. They were identified using 1000 Genomes Phase 3 projected principal components of population structure, retaining only those within three standard deviations from the mean for the top 100 principal components.”

* pg 15, line 312 'logistic regression was used...', it's not clear why this is
under the 'Estimating prediction accuracy' subsection, and how does it apply to
these PRS methods? Most of the PRS methods assume a linear regression model even
for binary outcomes.

*Response:*

This sentence is not referring to the PRS methods. It is stating that when we modelled the PRS to determine their association with phenotypes, we used a logistic regression for binary outcomes, and linear regression for continuous outcomes. We have clarified this point as follows:

“When modelling the polygenic scores, logistic regression was used for predicting binary outcomes, and linear regression was used for predicting continuous outcomes.”

* Which version of LDpred was used?

*Response:*

Originally, LDPred1 was used. In the revised version we have also included LDPred2.

* pg 5, line 63: 'Although genetic information is used to predict rare Mendelian
genetic disorders' -> most of the time rare Mendelian genetic disorders don't
need prediction since they manifest early in life, are highly penetrant, and
testing usually occurs after clinical presentation and not before. Perhaps
BRCA1/2 is the one standout example you can use here.

*Response:*

I agree. Thank you for the suggestion. This example has now been added as follows:

“Although genetic information is used to predict rare Mendelian genetic disorders, such as breast cancer based on *BRCA1/2* variants, our ability to predict common disorders using genetic information is currently insufficient for clinical implementation.”

* pg 6, line 98: 'Effect sizes estimated in a GWAS are typically larger than
they would be in an independent sample due to overfitting or winner’s curse'.
This text could use some clarification as it could confuse readers; in standard
GWAS, there is no overfitting (one predictor with thousands of samples) but due
to p-value selection there is a winner's curse which leads to inflation of
effect sizes for the statistically significant SNPs. In contrast, in
multivariable models (e.g., BLUP or lasso) there can be overfitting since the
number of SNPs is typically much larger than the sample size.

*Response:*

Thank you for this clear explanation. We have updated this section as follows:

“Polygenic scoring methods can lead to overfitting of genetic effects due to the p-value based selection of variants or joint estimation of many genetic effects.

To avoid this overfitting, genetic effect size estimates can be reduced using…”

* It's quite confusing to call one result '10FCVal' and another 'MultiPRS' when
the MultiPRS also used 10-fold cross-validation.

*Response:*

It is true that 10-fold cross validation is a part of the MultiPRS approach as well. However, we clearly describe the meaning of these terms throughout and so we have retained these terms.

* pg 24, line 497, much more salient examples of MultiPRS (aka metaGRS)
improving predictive power over single PRS are
https://www.onlinejacc.org/content/72/16
[https://www.nature.com/articles/s41467-019-13848-1](about:blank)

*Response:*

Thank you for highting these studies use of multiPRS. I have added reference to [https://www.onlinejacc.org/content/72/16](about:blank).

* Table 2: It's not quite right to say that e.g., LDpred needs only 'direction
of effect' as variant level data. LDpred v1 uses the (two-sided) p-value to
back-calculate the unsigned z-statistic, and it needs the effect direction to be
able to infer the sign of the original (signed) z-statistic. So it basically
needs both beta and the p-value.

*Response:*

Agreed. The same information is required for all methods, and I think this column might be misleading. We have removed this column from the table.

* Discussion, pg 23 'Then prediction is the aim and inference is not of
interest...': this paragraph is confusing. Adjusting PRS for PCs doesn't affect
whether prediction is the aim or not, but affects interpretation. If there is
substantial population stratification in the GWAS and/or target population that
is also correlated with the phenotype of interest, the analysis needs to be very
careful due to potential for confounding. Arguably, we don't want PRS for T2D
being good at prediction just because they predict people's ancestry which is
confounded with socioeconomic status. It does get rather blurry for phenotypes
like educational attainment.

*Response:*

Good point. I have now included a sensitivity analysis showing the relative performance of methods after PCs are controlled for. Therefore, I have removed this limitation/future direction from the discussion.

***Reviewer #3:***

Pain et al present a well written and a very timely benchmark study for polygenic risk score methods. In recent years a number of different methods have been proposed to derive polygenic risk scores based on GWAS summary statistics, and almost every paper finds that their proposed method is somehow the “best”. As a user, it is therefore hard to understand what method to use for a given dataset. Therefore benchmark studies like these are particularly timely and important. However, I feel that the benchmarks provided here do not necessarily provide a full picture of the field. First, not all published methods are considered in the benchmark, although some of the most commonly used are included. Second, only about a dozen phenotypes (diseases and traits) are used for the benchmark, most of which are known to be highly polygenic. The results may therefore not generalise well for less polygenic traits, such as some types of cancer, metabolic measurements, and gene expression values. Besides these issues, I generally enjoyed the paper, and I really liked the accompanying GenoPred webpage. I list these and other comments below.

1. A number of methods have been published in recent years, which may be of interest. E.g. AnnoPred (Hu et al., PLoS Comp Biol 2017), SCT (Privé et al., AJHG 2019), NPS (Chun et al., AJHG 2020), and DBSLMM (Yang and Zhou, AJHG 2020). I realize many of these have only been published recently, but I would appreciate it if the authors included at least a couple of these in their comparison. Other methods that have been proposed, but are at the pre-print stage, are LDpred2 (Privé et al., bioRxiv), ldpred-funct (Marquez-Luna et al., bioRxiv 2020), MegaPRS (Zhang et al., bioRxiv 2020). Although it would be nice to include some of these in the comparison, I do not really expect the authors to do that, since these are not published yet.

*Response:*

We agree it is important to compare a wider range of methods. As a result, we have provided a richer comparison by including comparison with DBSLMM and LDPred2. We have included discussion of the other methods suggested by the reviewer. We have not included MegaPRS as the associated study is not yet published. We have not included AnnoPred, or ldpred-funct, as these methods include functional annotations when deriving the polygenic score and are considered somewhat distinct from other methods. We have not included NPS or SCT as these methods are again somewhat distinct as they are unable to generate a polygenic score without a training sample with the desired phenotype. Furthermore, previous comparison of NPS and SCT indicates these methods are unlikely to improve prediction over the other methods included ([https://academic.oup.com/bioinformatics/advance-article/doi/10.1093/bioinformatics/btaa1029/6039173#221959715](about:blank#221959715), [https://www.sciencedirect.com/science/article/pii/S0002929720301518](about:blank)) .

2. Most or all of the traits/diseases considered here are highly polygenic. I recommend including a couple of more simple traits, as I am curious to understand what methods can predict those. These include blood and metabolite measurements, prostate cancer, breast cancer, or something like hair color, etc. Alternatively, you can examine the performance on different genetic architectures using simulations.

*Response:*

Thank you for this suggestion. We have now included two of the suggested less polygenic outcomes: prostate cancer and breast cancer.

3. In my experience, QCing the GWAS summary statistics and the LD reference is really important in practice, especially for MCMC approaches such as PRScs, LDpred, and SBayesR. It is unclear to me how robust the different softwares are to data issues. I know some software provide a set of “good” variants, such as PRScs and LDpred2, for which they also provide a LD reference. Other software requires the user to QC the data beforehand. I would be interested in understanding what methods are more robust to data issues, such as poorly tagged LD, vastly different sample sizes for variants, duplicated variants, etc. Perhaps you can add a simulation where you perturb the data a bit, or at least provide an overview of how different methods deal with these issues.

*Response:*

I agree this is an important consideration. Simulations go beyond the scope of this manuscript, but feel our study already reflects the sensitivity of these methods to quality control issues. We show that most methods are robust for a range of GWAS after some standard GWAS quality control. We have made the GWAS summary statistics QC protocol publicly available to help ensure future users of these methods perform sufficient quality control.

4. If some methods are more prone to population stratification, adjusting for PCs can affect the relative results. I would be interested in seeing the relative comparison between methods before and after adjusting for PCs.

*Response:*

Thank you for raising this point. To address this, we have added a sensitivity analysis in which the top 20 PCs have been regressed from polygenic scores in advance. We describe this addition in the methods and results sections:

Methods: “To determine whether certain methods are more prone to capturing genetic effects driven by population stratification, we carried out a sensitivity analysis, in which the first 20 principal components were regressed from the polygenic scores in advance. Principal components were derived in the 1KG Phase 3 reference, and then projected into UKB and TEDS samples.”

Results: “Controlling for the first 20 genetic principal components did not affect the relative performance of polygenic scoring methods (Figure S12).”

5. Can you please provide some statistics for the running times of the different methods.

*Response:*

Thank you for this suggestion and we agree this is an important consideration. We have included a Figure S13, comparing the time taken for each method to process GWAS summary statistics for chromosome 22 without the use of parallel functionality. We chose this design to avoid our implementation of the methods affecting the results, whilst reducing the runtime of each method. We describe this addition in the methods and results sections:

Methods – Runtime Comparison: “To compare the time taken for each polygenic scoring method to process GWAS summary statistics, we ran each method using GWAS summary statistics restricted to variants on chromosome 22. No parallel implementations were used in this comparison. Apart from LDPred1, all the polygenic scoring methods can be implemented in parallel.”

Results – Runtime Comparison: “The runtime of methods to process GWAS summary statistics on chromosome 22 without parallel implementations varied substantially (Figure S13). The methods (fastest to slowest) were pt+clump (~3 seconds), DBSLMM and lassosum (~30 seconds), SBLUP (~1 minute), SBayesR and LDPred (~3-6 minutes), PRScs (~35 minutes), and LDPred2 (~50 minutes). The number of parameters tested by each method will influence the runtime. For example, using only one shrinkage parameter for PRScs will take 1/5 of time taken for PRScs to use 5 shrinkage parameters.”

Figure S13. Runtime for each polygenic scoring method using genetic variants on chromosome 22. No parallel implementations were used.

6. Regarding the SBayesR, the results look weird as they suggest much worse performance than reported by the recent bioRxiv publications listed in point 1, and shown in Ni et al. (medRxiv 2020). Can you please elaborate on why this is the case.

*Response:*

We agree this discrepancy is notable and interesting. We worked with the SBayesR developers to try to resolve the problem, without success. We have added the following point to the discussion:

“Our study highlighted the performance of SBayesR is highly variable across GWAS summary statistics and on average does not perform well compared to other methods. In contrast, a recent preprint comparing polygenic scoring methods using depression and schizophrenia GWAS reports that SBayesR is the best approach [16]. This apparent discrepancy can be explained by our study testing methods across a wider range of GWAS. Indeed, for the one phenotype tested in both studies (depression), the results are highly concordant, with SBayesR performing better than other methods when using larger LD-reference datasets. Our study highlights the importance of validating methods based on GWAS for a range of phenotypes and from different discovery samples/consortia.”

7. 1KG is likely too small of an LD-reference for LDpred, SBLUP or SBayesR, which can affect relative performance. (As far as I can see, it is also not recommended.)

*Response:*

As the reviewer says, using reference samples that are larger than the 1KG reference are sometimes recommended by the newer polygenic scoring methods. This is why we also included the 10K European reference from the UK Biobank sample, and the GCTB-provided LD reference for SBayesR. In practice, we found the results were very similar across the different reference samples.

8. Also, SBLUP and LDpred-inf are two different implementations of the same method/idea (see e.g. Ni et al. (medRxiv 2020)).

*Response:*

Yes, this is interesting. In line with this point, the results between SBLUP and LDPred-inf are highly concordant in our study. In the revised manuscript, we highlight this point more clearly.

”Another approach to derive polygenic scores is to assume an infinitesimal model, as is done by SBLUP and the infinitesimal models of LDPred1 and 2 [16].”
